# Sex Differences in the Relationship of Biomarker Change to Memory Decline in Early Alzheimer’s Disease: an Observational Cohort Study

**DOI:** 10.21203/rs.3.rs-7661592/v1

**Published:** 2025-10-08

**Authors:** Erin E. Sundermann, Sarah J Banks, Mark W. Bondi, Maricedes Acosta Martinez, Anat Biegon, Lindsay J. Rotblatt, Thomas Hildebrandt

**Affiliations:** University of California, San Diego; University of California, San Diego; University of California, San Diego; Stonybrook University; Uppsala University; University of California, San Diego; Mt. Sinai School of Medicine

**Keywords:** Sex/gender, Alzheimer’s disease, mild cognitive impairment, preclinical Alzheimer’s disease, Alzheimer’s pathology, verbal memory, clinical presentation, cognitive reserve

## Abstract

**Background:**

Alzheimer’s disease (AD) exhibits sex differences in pathology and cognitive trajectories. Understanding how these differences manifest across the Alzheimer’s continuum can improve early detection, diagnostics, and interventions. We examined sex differences in how cerebrospinal fluid pTau181/Aβ42 ratio changes relate to verbal memory decline across the preclinical and mild cognitive impairment (MCI) stages of AD.

**Methods:**

In this retrospective, longitudinal, observational study, data were extracted from 404 participants (age range: 55–87.8, 98% non-Hispanic White) of the Alzheimer’s Disease Neuroimaging Initiative cohort study who were classified as either preclinical AD (69 females, 68 males) or MCI (113 females, 151 males) at baseline and had CSF pTau181/Aβ42 ratio and cognitive assessment data at at-least two timepoints. Using regression models, we examined the relationship between changes in CSF pTau181/Aβ42 and verbal memory and the moderating role of sex and AD stage over a mean follow-up period of 4 years. Verbal memory was represented by a composite z-score averaging Immediate and Delayed Recall z-scores of the Rey Auditory Verbal Learning Test. Covariates included baseline age, education, and apolipoprotein E genotype.

**Results:**

A significant sex x diagnostic group x biomarker change interaction (β = −17.47, 95%CI = 27.60 to −7.33, *p* = .001) indicated that sex differences in the relationship between changes in CSF pTau181/Aβ42 ratio and verbal memory differed by disease stage. While males in the preclinical AD stage showed steeper memory decline than females with increasing pTau181/Aβ42 ratios, this difference was not statistically significant. In contrast, in the mild cognitive impairment stage, a significant sex X biomarker change interaction (β = 10.17, 95% CI = 4.94 to 15.40, p < .001) in the MCI stage indicated that females exhibited significantly steeper memory decline associated with increasing pTau181/Aβ42 ratios compared to males.

**Conclusion:**

Sex differences in the relationship between AD biomarker levels and cognitive decline vary by disease stage. Although not statistically significant, females demonstrated resilience to memory decline in the preclinical stage, whereas, in the MCI stage, they experienced significantly steeper memory loss compared to males. Results suggest that accounting for sex in biomarker-based methods of disease detection and tracking can improve early detection and intervention in both sexes.

## PLAIN ENGLISH SUMMARY

Alzheimer’s disease (AD) affects men and women differently, but the reasons why remain unclear. Women are more likely to develop AD and often show higher levels of tau, a protein linked to brain changes in the disease. At the same time, women tend to have better memory than men in the very early stages, which may delay diagnosis until the disease has progressed further.

In this study, we analyzed data from 404 older adults in the Alzheimer’s Disease Neuroimaging Initiative. Participants were either in the “preclinical” stage of AD (biomarker changes but no memory problems) or had mild cognitive impairment (MCI). We measured changes in spinal fluid proteins (pTau181/Aβ42 ratio) linked to AD and tracked how they related to changes in memory performance over about four years.

We found that sex differences depended on the stage of disease. In the preclinical stage, men tended to show faster memory decline than women as AD biomarkers worsened, although this was not statistically significant. In contrast, among people with MCI, women showed significantly faster memory decline than men as biomarkers increased.

These findings suggest that women may be more resilient to early Alzheimer’s-related brain changes but reach a “tipping point” at which their memory declines faster once symptoms begin. Considering sex when interpreting AD biomarkers may improve early detection, diagnosis, and treatment for both men and women.

## BACKGROUND

The two-fold higher prevalence of Alzheimer’s disease (AD) in females is well-established [1]. Alzheimer’s disease is characterized by the hallmark pathologies of amyloid-beta plaques (Aβ) and phospho-Tau (p-Tau)-based neurofibrillary tangles followed by neurodegeneration. These pathologies form the basis of the A/T/N framework used for diagnosis and staging through biomarkers of Aβ deposition, tau pathology, and neurodegeneration. Many studies have examined sex differences in the burden of AD pathology and generally find that females typically show a greater burden of pathological tau than males, particularly among amyloid positive individuals who are either cognitively normal or have mild cognitive impairment (MCI) [2–6]. However, how the clinical presentation of AD pathology may differ for males and females is a question far less examined, although equally important to understand in terms of improving early detection, intervention and disease tracking in each sex.

Evidence so far suggests that, despite the greater tau burden in females compared to males, females tend to have a cognitive advantage over men in the preclinical AD stages (i.e., cognitively normal but AD biomarker positive) to early MCI stage of the AD continuum, particularly on tests of verbal memory [7–12]. Studies specifically probing the preclinical AD stage found little to no difference in verbal memory performance between PET-derived Aβ biomarker positive versus negative cognitively normal females, whereas verbal memory performance was significantly worse in Aβ biomarker positive versus negative, cognitively normal males (Caldwell et al., 2017; Caldwell et al., 2019). However, among individuals with MCI, females tend to show more rapid decline than males [13–15]. This evidence stems from a mix of cross-sectional studies examining sex differences in cognition at different diagnostic stages or pathology levels (mild, moderate or severe) and longitudinal studies of cognitive decline by diagnostic group. The cross-sectional studies have generally found that females show significantly better memory performance than males among those diagnosed as cognitively normal or MCI or among those with mild to moderate levels of AD biomarkers in the brain (i.e., hippocampal volume [16], Aβ [8, 11], tau [8, 9], and brain glucose metabolism [17]), whereas this sex difference was absent among patients with AD demntia or when biomarker levels were high [8, 9, 11, 16, 18, 19]. This pattern of results suggests a steeper memory decline in females in comparison to males when progressing from mild to moderate-severe pathological burden. Longitudinal studies have supported the steeper decline in females at intermediate disease stages as measured by clinical symptom severity or more advanced pathology burden [13–15].

This pattern of sex differences in the clinical trajectory and clinical presentation of AD pathology suggests sex differences in the relationship between AD pathology and clinical symptoms and that this difference varies depending on the stage of the AD continuum. However, longitudinal studies have not yet investigated the effects of sex on this progression.

Here we examined sex differences in the relationship of changes in cerebrospinal fluid (CSF) pTau_181_/Aβ_42_ ratio to changes in verbal memory and the moderating role of disease stage among Alzheimer’s Disease Neuroimaging Initiative (ADNI) participants classified as Preclinical AD or MCI at baseline. We chose this marker rather than examining Aβ and tau biomarkers individually because the ratio captures the interplay between Aβ and tau pathologies and this interplay is more closely tied to disease progression and cognitive decline [20–22] and shows critical sex differences [4]. We hypothesized that increases in the pTau_181_/Aβ_42_ ratio are associated with greater memory decline in males relative to females among Preclinical AD individuals but the opposite is true in MCI individuals, in whom increases in the pTau_181_/Aβ_42_ ratio are associated with greater memory decline in females compared to males.

## METHODS

### PARTICIPANTS AND DATA SOURCE

Data collected between 2005 and 2019 were extracted from the ADNI database. ADNI data is publicly available at adni.loni.usc.edu. ADNI is a longitudinal, multi-site, cohort study that began in 2003 as a public-private partnership and recruited a cohort of healthy older adults (age ≥ 55), and individuals with MCI and early AD. Information about ADNI can be found at www.adni-info.org. ADNI study visits involve neuroimaging, neuropsychological and clinical assessments. The general enrollment inclusion/exclusion criteria for ADNI have been described elsewhere [23]. This specific study included participants who met the following inclusion criteria: (1) classified as either preclinical AD or MCI at baseline; (2) had CSF AD biomarker and cognitive assessment data at baseline as well as at-least one follow-up visit; (3) had relevant covariate data at baseline (e.g., demographics, apolipoprotein E ε4 [APOE-ε4] status. Of the 704 individuals classified as preclinical AD or MCI at baseline, a sample of 404 older individuals (age range: 55–88) classified as preclinical AD (69 females, 68 males) or MCI (113 females, 151 males) at baseline had available biomarker and covariate data.

### DIAGNOSTIC CLASSIFICATION

Diagnosis of cognitively normal versus MCI was based on the Jak/Bondi classification method [24]. Applied to ADNI, the Jak/Bondi diagnosis includes six neuropsychological tests representing three cognitive domains: Trail-Making Tests A and B (psychomotor speed/executive function), Category Fluency and Boston Naming Test (language) and the RAVLT Delayed Recall and Recognition Tests (episodic memory). The normative regression procedure incorporated age, sex, and education to account for their influence on cognitive performance. An impaired score was defined as > 1 SD below the age-, sex- and education-corrected normative mean. MCI diagnosis was provided if the participant did not meet diagnostic criteria for dementia based on the standard NINCDS/ADRDA criteria [25] and met one of two of the following criteria: 1) impaired score on two tests within a cognitive domain or 2) one impaired score in each of the three cognitive domains. Participants who did not meet one of these two criteria were classified as cognitively normal. Among cognitively normal individuals, those who were positive for either CSF p-Tau/Aβ ratio, amyloid PET or Tau PET biomarkers based on established cut-points [26–28] were classified as preclinical AD.

### COGNITIVE OUTCOME

ADNI study visits include a battery of neuropsychological tests. We chose episodic memory as our outcome of interest because tests of verbal memory are most often used in MCI/AD diagnostic protocols. Episodic memory was measured via a verbal memory test, the Rey Auditory Verbal Learning Test (RAVLT). The RAVLT involves learning a list of 15 unrelated words and recalling as many words as possible across five immediate recall trials (“Immediate recall”, range: 0–75), after learning an interference list, and after a 30-minute delay period (“Delayed Recall score”, range: 0–15). The Immediate and Delayed Recall scores were z-scored and then averaged together to create a verbal memory composite score. The RAVLT has previously shown a strong female advantage [29]. Change score was calculated by creating lagged residuals with a lag of one time point.

### CSF AD BIOMARKER

We examined the ratio of CSF levels of hyperphosphorylated tau-181 (p-tau181) to Aβ1–42 proteins (pTau_181_/Aβ_42_ ratio). To model change in biomarker levels, we generated lagged residuals by predicting each biomarker value from its value at the prior visit (lag of one time point). The residuals from these models—representing deviation from the expected value based on prior levels—were then used as the measure of biomarker change. This approach reduces bias from autocorrelation in repeated measures.

### STATISTICAL ANALYSES

Differences in sample characteristics and variables of interest at baseline between sexes were examined using independent t-tests for continuous variables and Chi-square tests for categorical variables. Regression models were built using lme4 package [30] in R v4.1.3 [31] to test for the effect of change in CSF pTau_181_/Aβ_42_ biomarker levels on the verbal memory composite score. To reduce potential sources of sampling bias, we included age, education, and income in regression models.

The regression models also adjusted for APOE-ε4 carrier status. In the overall sample, we tested for the 3-way interaction of sex*biomarker change*group (MCI vs. Preclinical AD). We then examined interaction effects of sex*biomarker change in separate models for those with MCI and those in the preclinical stage. We conducted a sensitivity analysis restricting the MCI group to those who are AD biomarker positive (CSF p-Tau/Aβ ratio, amyloid PET or Tau PET biomarkers) in order to increase the likelihood that the MCI individuals are on the AD trajectory as MCI is a heterogenous condition and to better align them with the preclinical AD group (defined by biomarker positivity) but representing a more advanced stage characterized by mild cognitive deficits. Missing data were not replaced. Only individuals with complete data were included in the sample.

## RESULTS

Table 1 summarizes sex differences in sample characteristics at baseline. Males were significantly more educated, had significantly lower memory scores and a significantly higher proportion of MCI cases than females. The average CSF pTau_181_/Aβ_42_ ratio and the frequency of the APOE-ε4 carrier status did not significantly differ between sexes. The average follow-up duration for both males and females was about 4 years after baseline.

In the full sample, the sex* biomarker change*Group (Preclinical vs. MCI) interaction was significant (β = −17.47, 95%CI = 27.60 to −7.33, *p* = 0.001). Table 2 summarizes the full model. Evidence of the significant 3-way interaction indicates that the relationship between change in the pTau_181_/Abeta_42_ ratio and change in the verbal memory composite score significantly differed by sex and group status. The greater influence of change in the pTau_181_/Aβ_42_ ratio on change in verbal memory composite score was evident in the MCI group only, controlling for covariate effects.

**Figure 1:**
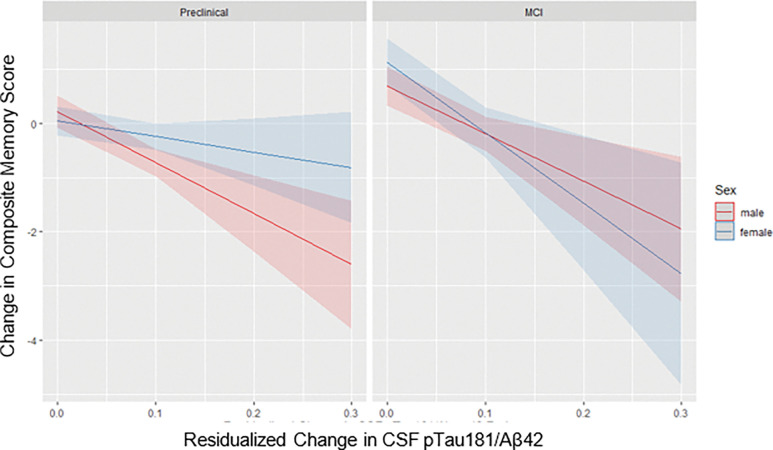
Relationship between change in pTau181/Aβ42 ratio and change in memory by sex and diagnostic group. Relationship between change in pTau181/Aβ42 ratio and change in the verbal memory composite score by sex and baseline diagnostic group. Males are in red and females are in blue. Those who were classified as preclinical at baseline are pictured in the left panel and those classified as MCI at baseline are pictured in the right panel. The average follow-up duration for both males and females is approximately 4 years.

Results from regression models separated by preclinical AD and MCI status are in Table 3 and visually depicted in Fig. 1. In the preclinical group, change in pTau_181_/Abeta_42_ ratio was not a significant predictor of change in the verbal memory composite score (β = −9.10, 95%CI = −34.61 to 16.41, *p* = 0.48). The wide confidence interval indicates a lack of precision in the measured effect between these two change processes. Although the pattern of results was as hypothesized (i.e., females showing less memory decline than males as pTau_181_/Aβ_42_ increased), the interaction (β = −3.73, 95%CI = −22.78 to 15.31, *p* = 0.70) or the main effect of sex (β = −0.10, 95%CI = −0.27 to 0.08, *p* = 0.28) were not significant. In contrast, the MCI model demonstrated a different pattern of results. There was a significant negative relationship between change in the pTau_181_/Aβ_42_ ratio and change in the verbal memory composite score (β = −23.80, 95% CI −32.78 to −15.18, *p* < 0.001). There was also a significant main effect of sex indicating a significantly steeper decline in the verbal memory composite score among females (β = −0.10, 95%CI = −0.17 to −0.03, *p* = 0.009). Most notable, a significant sex * biomarker change interaction (β = 10.17, 95% CI = 4.94 to 15.40, *p* < 0.001) indicated that females experienced a stronger influence of change in the pTau_181_/Abeta_42_ ratio than males on their decline of the verbal memory composite score.

In sensitivity analyses limiting the MCI sample to those who are AD biomarker positive, the results didnot change.

## DISCUSSION

This is the first longitudinal study to demonstrate that sex differences in the clinical presentation of AD pathology may differ between the preclinical and prodromal stages of AD. In line with hypotheses, we found that males in the preclinical AD group showed steeper memory decline as AD pathology advanced compared to females in the preclinical AD group, but this difference was not statistically significant perhaps due, in-part, to the smaller sample size and consequent lower statistical power and greater variability in the preclinical AD group. The pattern of results in the MCI group also align with hypothesis in that females showed significantly steeper memory decline as AD pathology advanced compared to males. This finding suggests that females with MCI experience a steeper decrease in memory function per unit of increasing CSF pTau_181_/Aβ_42_ ratio than males and aligns with past studies reporting greater cognitive decline in females compared to males with MCI in general. Overall, the pattern of results suggests that females are better able than males to maintain normal memory performance despite early AD pathogenesis; however, there seems to be a tipping point, whereby, when AD pathogenesis reaches more advanced stages, it has a more detrimental impact on memory decline compared to males.

The pattern of a female memory advantage in preclinical stages followed by faster decline in later stages has been suggested by prior cross-sectional studies showing a memory advantage in females among those with mild AD biomarker burden (i.e., Aβ, tau, hippocampal volume, brain glucose metabolism) that disappeared among individuals with moderate-to-severe pathological burden, suggestive of a steeper decline in females [8, 9, 11, 16, 18, 19] Studies by Caldwell et al. specifically examined the influence of sex at the preclinical AD stage. They found significantly worse verbal memory performance in cognitively normal males who were PET-derived Aβ biomarker positive versus biomarker negative, whereas this difference was absent in females [7, 8], suggesting that memory function in females is more resilient to the adverse effects of early-stage AD pathogenesis, resulting in less decline at this stage.

Longitudinal studies have supported the steeper decline in females at later disease stages as measured by clinical diagnosis (i.e., MCI) or advanced pathology. Previously in ADNI, Lin et al. found two fold faster decline in females with MCI compared to males with MCI on the global cognitive tests of the Alzheimer’s Disease Assessment Scale–Cognitive Subscale (ADAS-COG) and the Clinical Dementia Rating-Sum of Boxes (CDR-SOB) [15]. In 688 ADNI participants, Holland et al. compared sex/gender differences in cognitive and brain volume decline among cognitively normal, MCI and AD dementia groups [14]. They found that, despite females showing steeper declines in hippocampus, entorhinal cortex and amygdala volumes in the cognitively normal group, steeper cognitive decline (ADAS-Cog and CDR-SOB performance) in females was only found in the MCI and AD dementia groups [14]. This pattern of results again suggests memory performance that is more resilient to early AD pathogenic changes in females compared to males, whereas the opposite is true in later stages. Our current study is a much-needed next step in this literature by demonstrating longitudinally that how memory function is affected by advancing AD pathology depends on sex and that these sex differences are not uniform across the AD trajectory.

Our results are also consistent with recent findings from Memel et al. (2025) in a large autosomal dominant frontotemporal dementia (FTD) cohort from the ALLFTD Consortium, where females showed cognitive resilience in asymptomatic stages followed by more rapid decline than males in symptomatic stages, despite similar levels of neurodegeneration markers early on [32]. This parallel pattern across two distinct neurodegenerative diseases suggests that such stage-dependent sex differences in clinical trajectories may not be AD-specific, but rather may reflect broader, sex-related mechanisms of resilience and vulnerability in the face of advancing neurodegeneration.

Sex differences in the clinical presentation of AD pathology have critical research and clinical implications. One such implication is the vital role of AD biomarkers in early detection and disease tracking that is swiftly expanding as technology advances [33]. Biomarker research and clinical use has, for the most part, operated under the assumption that biomarkers and their cut-points reflect similar disease risk, stage and progression in males and females. Diagnostic thresholds for biomarkers and our theoretical models of the temporal progression of biomarkers will likely be improved if sex-specific patterns are accounted for. Understanding sex differences in the link between AD biomarkers and clinical trajectories and incorporating sex-specific considerations into guidelines for biomarker-based tools can lead to earlier and more accurate diagnoses, improved disease monitoring and better-targeted interventions for both males and females. Other critical implications revolve around evidence from the current and past findings suggesting that females are at a more advanced disease stage than males, pathologically, when diagnosed with MCI [10, 11, 17, 34]. This results in limited opportunities for early diagnosis and intervention in females when our currently-available pharmaceutical and non-pharmaceutical interventions have the greatest potential of altering the disease course. Lastly, the differing pattern of sex differences by disease stage indicates the importance of accounting for disease stage when conducting research examining sex differences in AD. This is particularly true for cross-sectional studies, in which sex difference findings may be masked when examined across preclinical, MCI and AD dementia stages.

We can only speculate as to the reasons behind the sex differences in the clinical presentation of AD pathology. They are likely multi-factorial but one critical factor is the life-long female advantage in verbal memory. The higher baseline verbal memory performance in females means they have further to fall on the AD trajectory. In combination with female’s purported delayed onset of verbal memory decline until a later disease stage, this results in a more intense and steeper decline in females during the MCI to dementia transition. There may be biological mechanisms that support female’s cognitive resilience to early AD pathology followed by their steeper decline thereafter. For instance, greater brain glucose metabolism in females compared to males that is sustained at early, but not later, AD stages could potentially provide greater resilience against early AD-related brain changes [35–40]. Additionally, in a post-mortem study examining translocator protein (TSPO) levels in brain tissue as a marker of neuroinflammation, Acosta-Martinez et al. found that, among females but not males, there were significant, region-dependent elevations of TSPO density in AD dementia cases compared to MCI and cognitively normal cases and a significant, positive correlation between TSPO density and tau burden [41]. A stronger neuroinflammatory response in females with AD relative to males was also reported recently by Biechele et al. [42]. These findings raise the possibility that increases in neuroinflammation later in the AD trajectory may contribute to female’s steeper cognitive decline.

Our study has limitations. ADNI is a convenience sample of mostly white and well-educated volunteers, which limits generalizability of results. It is imperative to examine this research question in more diverse samples that better reflect the U.S. population and understand how social determinants of health influence sex/gender differences in AD. It would be informative to repeat our analyses with a visual memory test to see how results compare with a memory task that does not show a sex bias; however, this data is unavailable in ADNI. Lastly, our sample size and, in turn, statistical power was limited once stratifying by diagnostic group, particularly in the preclinical group where the sex difference pattern was as hypothesized but not statistically significant.

## CONCLUSIONS

In conclusion, we found that sex differences in how changes in biomarkers of AD pathology relate to verbal memory decline vary by disease stage. Although the moderating role of sex was not significant in the preclinical AD group, the pattern of results suggests that females showing less memory decline than males in response to advancing AD pathology. However, in the MCI stage, the sex difference was in the opposite direction, whereby females showed significantly steeper memory decline than males in response to advancing AD pathology. While this change in sex disparities in the AD pathology and symptomology link was suggested by prior cross-sectional studies, this longitudinal study represents a more definitive test of this hypothesis. Overall, the change in sex differences highlights a possible “tipping point” in females, where resilience transitions to vulnerability as the disease progresses. These findings have critical clinical and research implications. They emphasize the need for sex-specific approaches to AD biomarker-based diagnostics and interventions to improve outcomes for both sexes, especially in early AD detection in the higher risk sex, females.

## Figures and Tables

**Table 1:** Sample characteristics at baseline by sex.

	Females (N = 182)	Males (N = 219)	p-value
Age, M (SD)	73.6 (6.0)	74.9 (7.0)	.20
Years of education, M (SD)	15.3 (2.6)	16.6 (2.7)	**.002**
Race, % non-Hispanic white, N (%)	178 (97.7%)	212 (96.7%)	.84
APOE-ε4 carrier, N (%)	107 (59.0%)	121(55.2%)	.67
Diagnostic Group			.03
Preclinical AD, N (%)	78 (43.0%)	73 (33.3%)	
MCI, N (%)	104 (57.0%)	146 (66.7%)	
Follow-up period (yrs), M (SD)	4.0 (2.4)	4.4 (2.8)	.17
Progressed to dementia, N (%)	61 (33.3%)	81 (37.1%)	.26
Baseline CSF pTau_181_/Aβ_42_ ratio, M (SD)	0.045 (.031)	0.042 (0.024)	.32
Baseline verbal memory composite score, M (SD)	0.02 (0.89)	-0.74 (0.78)	**.004**
CSF pTau_181_/Aβ_42_ change score, M (SD)^a^	0.055 (0.033)	0.053 (0.024)	.42
Verbal memory composite change score, M (SD)^a^	0.116 (0.962)	-0.123 (0.887)	**.003**

Notes. AD = Alzheimer's disease, MCI = mild cognitive impairment, pTau = hyperphosphorylated tau, Aβ = amyloid-beta, APOE-ε4 = apolipoprotein E ε4 allele.

a Higher absolute value indicates greater change, while negative values indicate decline and positive values indicate improvement.

**Table 2:** Moderated effects of diagnostic group on Sex * pTau181/A42 change effects on change in memory performance.

β			
	Memory Composite Z-Score		
Predictors	Estimates	95% CI	*p* value
Intercept	0.75	0.22–1.29	**0.006**
pTau_181_/Aβ_42_ change score	−19.97	−28.35 - −11.58	**< 0.001**
Sex	−0.32	−0.63–0.00	0.051
Diagnostic group	−0.84	−1.72–0.05	0.064
Age (centered)	−0.01	−0.02–0.00	0.217
Years of education (centered)	0.01	−0.02–0.03	0.648
APOE-ε4 carrier status	−0.11	−0.25–0.03	0.114
pTau_181_/Aβ_42_ change score * Sex	8.05	3.28–12.81	**0.001**
pTau_181_/Aβ_42_ change score * Diagnostic group	22.91	7.54–38.29	**0.004**
Sex * Diagnostic group	1.05	0.48–1.61	**< 0.001**
(pTau_181_/Aβ_42_ change score * Sex) * Diagnostic group	−17.47	27.60 - −7.33	**0.001**
**Random Effects**			
σ^2^	0.56		
τ_00 ID_	0.12		
ICC	0.18		
N	402		

Note. CI = confidence interval, pTau = hyperphosphorylated tau, Aβ = amyloid-beta, APOE-ε4 = apolipoprotein E ε4 allele

**Table 3:** Comparison of diagnostic group models for change in memory performance

	Preclinical Group - Verbal Memory Composite Score			MCI Group - Verbal Memory Composite Score		
	Estimates	95% CI	p-value	Estimates	95% CI	p-value
Intercept	0.15	−0.15–0.45	0.322	0.07	−0.06–0.19	0.282
pTau_181_/Aβ_42_ change score	−9.10	−34.61–16.41	0.483	−23.98	−32.78 - −15.18	**<0.001**
Sex	−0.10	−0.27–0.08	0.282	−0.10	−0.17 - −0.03	**0.009**
Age (centered)	−0.01	−0.02–0.01	0.303	−0.00	−0.01–0.00	0.647
Years of education (centered)	0.02	−0.01–0.05	0.142	−0.01	−0.02–0.00	0.102
APOE-ε4 carrier status	−0.15	−0.31–0.02	0.083	0.04	−0.03–0.11	0.296
pTau_181_/Aβ_42_ change score * Sex	−3.73	−22.78–15.31	0.700	10.17	4.94–15.40	**<0.001**
**Random effects**						
σ^2^	0.31			0.10		
τ_00 ID_	0.05			0.02		
ICC	0.14			0.18		
N	138			265		
R^2^	0.194			0.266		

Note. MCI = mild cognitive impairment, CI = confidence interval, pTau = hyperphosphorylated tau, Aβ = amyloid-beta, APOE-ε4 = apolipoprotein E ε4 allele

## Data Availability

The dataset supporting the conclusions of this article is available in the ADNI data repository, [https://adni.loni.usc.edu/data-samples/adni-data/]. All ADNI data are shared through the LONI Image and Data Archive (IDA), a secure research data repository. Interested scientists may obtain access to ADNI imaging, clinical, genomic, and biomarker data for the purposes of scientific investigation, teaching, or planning clinical research studies. Access is contingent on adherence to the ADNI Data Use Agreement and the publication policies outlined in the documents available at https://adni.loni.usc.edu/data-samples/adni-data/.

## HIGHLIGHTS

- Alzheimer’s disease (AD) biomarkers in cerebrospinal fluid (pTau181/Aβ42) were linked to verbal memory decline differently in women and men, showing that sex modifies biomarker–cognition relationships across disease stages.
- Despite women showing more resilience to memory decline than men as the AD biomarker advanced in the preclinical stage, this pattern significantly reversed at the MCI stage, whereby women showed significantly faster memory decline than men as the AD biomarker advanced.
- Findings suggest a possible “tipping point” in women, where early resilience transitions to greater vulnerability as AD progresses.
- Results underscore the importance of considering sex when using biomarkers to detect, track, and treat AD.
